# Supplementary material for: Two time scales of adaptation in human learning rates
Source: eLife. 2026 Jul 22;14:RP108223. doi: 10.7554/eLife.108223 (PMC13391084; doi:10.7554/eLife.108223)
Supplement: Peer review history

# Editor report: eLife Assessment

**DOI:** 10.7554/eLife.108223.3.sa0

**Authors:** Andreea Oliviana Diaconescu

**Affiliations:** University of Toronto Canada

**Keywords:** Convincing, Valuable

This study makes a **valuable** contribution to the understanding of meta-learning and its neural mechanisms by distinguishing two timescales of learning rate adaptation: rapid, within-block reductions and slower, location-specific, meta-learned adjustments. Behavioural data and computational modelling provide **convincing** evidence that individuals adjust learning rates both rapidly in response to uncertainty and more gradually through meta-learning of environmental statistics. Neuroimaging results indicate that meta-learned learning rates are represented in orbitofrontal cortex, and that prediction errors are encoded across a distributed network including the ventral striatum, where they are modulated by expectations about error magnitude. The manuscript is timely and clearly written and opens the door to future work on how these signals contribute to adaptive behaviour.

# Referee report: Reviewer #1 (Public review)

**DOI:** 10.7554/eLife.108223.3.sa1

Summary:

Simoens and colleagues use a continuous estimation task to disentangle learning rate adjustments on shorter and longer timescales. They show that participants rapidly decrease learning rates within a block of trials in a given "location", but that they also adjust learning rates for the very first trial based on information accrued gradually about the statistics of each location, which can be viewed as a form of metalearning. The authors show that the metalearned learning rates are represented in patterns of neural activity in the orbitofrontal cortex, and that prediction errors are represented in a constellation of brain regions including ventral striatum, where they are modulated by expectations about error magnitude to some degree. The work opens the door to future work focusing on how exactly these signals contribute to adaptive behavior.

Strengths:

The authors build on an interesting task design allowing them to distinguish moment-to-moment adjustments in learning rate from slower adjustments in learning rate corresponding to slowly gained knowledge about the statistics of specific "locations". Behavior and computational modeling clearly demonstrate that individuals adjust to environmental statistics in a sort of metalearning. fMRI data reveal representations of interest including those related to adjusted learning rates and their impact on the degree of prediction error encoding in the striatum.

Weaknesses:

It was nice to see that the authors could distinguish differences between the OFC signals that they observed and those in the visual regions based on changes through the session. However, the linkage between these brain activations and a functional role in generating behavior remains somewhat unclear, opening the door for alternative interpretations.

Comments on revised version.

I appreciate the authors responses and they have largely addressed my concerns. I understand the concerns about power with regard to the individual differences/behavioral analyses included in the rebuttal. However, my personal view, which is perhaps a matter of taste, is that the paper would benefit from a description of these results - along with a clear description of why the authors are hesitant to draw a strong interpretation from the negative result.

# Referee report: Reviewer #2 (Public review)

**DOI:** 10.7554/eLife.108223.3.sa2

Summary:

Across two experiments, this work presents a novel spatial predictive inference paradigm that facilitates the investigation of meta-learning across multiple environments with distinct statistics, as well as more local learning from sequences of observations within an environment. The authors present behavioral data indicating that people can indeed learn to distinguish between noise levels and calibrate their learning rates accordingly across environments, even on initial trials when revisiting an environment. They complement their behavioral results with computational modeling, further bolstering claims of both local and global adaptation. Additional fMRI results support the role of OFC in this meta-learning process, with central OFC activity reflecting similarity between environments. This similarity emerges over time with task experience. Holistically, this paradigm and these data add to our understanding of how humans dynamically adapt their behavior on different timescales.

Strengths:

The novel paradigm represents a clever and creative expansion of spatial predictive inference tasks. The cover story was well chosen to facilitate an intuitive understanding of both the differences between environments, and the estimation of the mean within environments.

Additionally, the authors present complementary results from two experiments, which strengthens the behavioral findings. This is especially effective as the initial experiment's results were a bit noisy, and the modifications within the second experiment increased both power and the specificity/accuracy of participant predictions. Taken together, the behavioral results provide convincing evidence that participants did distinguish environments based on their underlying statistics and adapted their initial behavior accordingly.

Beyond this, the combination of behavioral results, computational modeling, and neuroimaging enhances the impact of the work. It paints a fuller picture of whether and how humans meta-learn the global statistics of environments, and this is an important direction for the field of adaptive learning.

Weaknesses:

Throughout much of the paper, the authors refer to the distinctions between environments primarily as differences in "initial learning rates" or "environment-specific learning rates." The optimal initial learning rate did indeed differ across environments -- the result of differences in underlying task statistics. These differences in task statistics result in distinct optimal initial learning rates and also vary with aspects of spatial position (e.g. vertical position in the example figure). The authors convincingly show that OFC activity increasingly reflects these variables throughout task experience. Given that these variables vary together, future work will be needed to distinguish whether particular variables drive these dynamics, or whether together they combine to evoke the representational differences.

The current work is also quite suggestive of meaningful individual differences in both local and global adaptive learning, in line with other prior work on predictive inference. This is perhaps underexplored in this data set, but certainly leaves the topic ripe for follow up going forward.

Finally, more information on all clusters that survived multiple comparisons correction would be useful, even in the absence of a priori hypotheses. For instance, there is commentary in the discussion section on the ACC, but this is not mentioned in the results, and it is unclear whether there were other undescribed clusters that survived correction.

# Author comment: Author response

**DOI:** 10.7554/eLife.108223.3.sa3

**Authors:** Jonas Simoens, Senne Braem, Pieter Verbeke, Haopeng Chen, Stefania Mattioni, Mengqiao Chai, Nicolas W Schuck, Tom Verguts

**Affiliations:** Ghent University Ghent Belgium; Ghent University Ghent Belgium; Howest University of Applied Sciences Kortrijk Belgium; Ghent University Ghent Belgium; Ghent University Ghent Belgium; Ghent University Ghent Belgium; University of Hamburg Hamburg Germany; Ghent University Ghent Belgium

The following is the authors’ response to the original reviews.

**Public Reviews:**

**Reviewer #1 (Public review):**

It was nice to see that the authors could distinguish differences between the OFC signals that they observed and those in the visual regions based on changes through the session. However, the linkage between these brain activations and a functional role in generating behavior was left unexplored. Without further exploration, it is hard to tell exactly what role the signals might be playing, if any, in the behavior of interest.

To link the behavioral with the fMRI data, we now correlated fMRI decoding accuracy with behavioral performance. We studied behavioral performance in two ways: the difference in high versus low noise environment learning rates, and mean accuracy (i.e., absolute prediction error). We correlated both measures with the decodability of the environment in the central OFC. Each correlation was calculated either in the full experiment, or only the second half. However, none of these correlations were significant (all *p* > .1). Given the difficulty of interpreting this result, and our lack of statistical power for doing individual difference analyses, we decided not to report these analyses in the final paper.

**Reviewer 2 (public review):**

(1) The authors make the distinction between meta-learned "global" learning rates and within environment learning rate adaptation in response to "local" fluctuations/observations. Though the experimental paradigm is novel, there are certainly links to prior work - for instance, though change point structures don't entail revisiting unique environments, they do require meta-learning from environmental statistics that is distinct from transient local adaptation to prediction errors. This tendency to increase one's learning rate after large prediction errors is appropriate in change point environments, though, as is true in this study, the amount of increase should be dependent on. This represents a similar kind of slower-timescale learning or reuse of more "global" parameters, and can be seen to different extents in prior work. It might benefit readers if the authors were to link the current work to previous research more explicitly to draw clearer connections between the approaches and findings.

We thank the reviewer for their very helpful literature suggestions and now contextualize and discuss our findings in light of relevant literature.

(2) Throughout much of the paper, the authors refer to the distinctions between environments primarily as differences in "initial learning rates" or "environment-specific learning rates." This is particularly prominent when discussing fMRI results. Though the optimal initial learning rate did differ across environments, this was the result of differences in underlying task statistics. It will be important to clarify this throughout the text, because of the confounds between task statistics and initial learning rate (and to some extent, the position on the screen), it is not possible to separate the impact of these specific variables. This is also relevant to understanding the justification for using methods like RSA to test whether brain regions represent task states similarly. If the main hypothesis is that neural activity reflects the (initial) learning rate itself, then a univariate analysis approach would seem more natural.

We agree that task statistics are not the same as differences in learning rates. However, we do not consider this as a confound: The point of the differences in task statistics is exactly to generate differences in learning rates. With our paradigm, we deliberately tried to dissociate variations in learning rate that were induced by learned environmental differences versus local task statistics. We tried to make this dissociation more clear, especially when discussing the fMRI results.

(3) For the neuroimaging results in particular, the specificity of some of the results (e.g. ventral striatum showing an effect of prediction error only in the low noise condition in the second half of task experience, only on the first trial) is a bit surprising. Additional justification of or context for these results would be useful to help readers gauge how expected or surprising these findings are.

We agree some of these findings were unexpected. We now also highlight that while we expected the ventral striatum to be involved in prediction error processing, we had no strong a priori expectations regarding these further modulations by time and environment. We also tried to contextualize these interactions more.

(4) There are some methodological details that are unclear (e.g., how were the positions of the crabs selected relative to the location they emerged from? Looking at Figure 1C, it looks like the crabs spread out unevenly, and that the single position they emerge from is not necessarily at the center of the crab locations.) Additional detail and clarity would help address some unanswered questions (more details below).

We clarified the experimental procedure at several places, and now added a video that helps illustrate the trial timeline better.

**Recommendations for the authors:**

**Reviewer #1 (Recommendations for the authors):**

(1) With regards to the primary weakness mentioned above, it would be nice to have some link between the brain signals of interest and upcoming behavior. For example, can you read something out of OFC that enables you to better predict what the participant will do next? Or even better, do so beyond any behavioral variability that is explained by the computational model?

To link the behavioral with the fMRI data, we now correlated fMRI decoding accuracy with behavioral performance. We studied behavioral performance in two ways: the difference in high versus low noise environment learning rates, and mean accuracy (i.e., absolute prediction error). We correlated both measures with the decodability of the environment in the central OFC. Each correlation was calculated either in the full experiment, or only the second half. However, none of these correlations were significant (all *p* > .1; see plots in Author response image 1). Given the difficulty of interpreting this result, and our lack of statistical power for doing individual difference analyses, we decided not to report this analysis in the paper.

**Author response image 1.**

(2) A number of the learning analyses are based on splitting the session into halves. As a first pass, this seems like a reasonable thing to do, but I certainly wonder what the dynamics of the meta-learning actually look like, and it seems like the data collected would be sufficient to gain some insight into those dynamics through some sort of sliding window analysis.

We thank the reviewer for this interesting suggestion, which was also raised by Reviewer 2. We now calculated the learning rate in a sliding window of 20 trials (i.e., trial *x* to *x* + 19), and provide revised figures for each experiment separately (Fig. 2E and Fig. 4E, respectively).

(3) The model selection procedures described make sense, but it would still be useful if the authors justified them by showing that they work in synthetic data (ie, generate a confusion matrix). I may be confused about what delta-SE is, but I'm confused about why two models with very different fits have the same value (211) for that metric.

We report model recovery on synthetic data, which yielded model recovery rates of 100%, and added these to our Methods section. To clarify the Reviewer’s second point, ∆SE is the standard error of the difference between a model’s LOOIC and the top ranked model’s LOOIC. There is no one-to-one mapping between the ∆SE and a model’s LOOIC.

(4) Was the central OFC anatomical ROI overlapping with the cluster surviving in the whole brain analysis? I didn't see this mentioned in the text, and it certainly would be important for interpreting the two results together.

The central OFC indeed overlapped with the cluster surviving whole brain analysis, which we report on page 17-18.

(5) The authors found regions that reflected learning rate at the "island presentation" phase of the task - it could be distinguishing this analysis and its meaning from other work that has focused on representations of learning rate at the time of feedback.

We agree that this is an important distinction worth emphasizing. Therefore, we added the following lines to our discussion paragraph:

“Importantly, previous studies examined neural correlates of learning rates during outcome evaluation, where learning rates may be adjusted online as a function of locally experienced prediction errors (e.g., Behrens et al., 2007; Browning et al., 2015; Nassar et al., 2012). In contrast, our RSA analysis targeted neural activity at island presentation, before any outcome information was available. At this moment, learning rates cannot be updated based on current feedback and instead reflected the retrieval of a previously learned, environment-specific learning-rate settings. This difference reflects our hypothesis that the OFC represents the latent states in a cognitive map of the task (Knudsen & Wallis, 2022; Moneta et al., 2024; Schuck et al., 2018; Wilson et al., 2014), which are expected to activate as soon as the agents can infer which task state it is in. Several studies have identified such “partially observable” task states in the medial OFC (Bradfield et al., 2015; Schuck et al., 2016; Tan et al., 2025; Wimmer & Büchel, 2019), in line with the region identified here (but see e.g., (Ongur & Price, 2000), for important anatomical distinctions between medial and lateral OFC and (Tan et al., 2025) for an example of related functions in lateral OFC). Our finding extends this notion by suggesting a link between OFC and meta learning, wherein meta-learned information becomes encapsulated in task states (Hattori et al., 2023; Moneta et al., 2024).”

(6) "Specifically, it showed a more negative response to larger (location) prediction errors, which is consistent with its documented role in showing a more positive response to more positive reward prediction errors (Calderon et al., 2021) - keeping in mind that being closer to the centre of where the crabs appeared (i.e., smaller location prediction errors) is less negatively or more positively surprising (i.e. smaller negative or larger positive reward prediction errors)."

I found this sentence very hard to parse. Do PE responses in the high noise environment get "compressed" in their representation over time (ie, it takes a larger error to get the same BOLD response)? If so, this relates to claims made in Diederen 2016... but see also Mah 2024 Cell Reports, who fails to see learning rate encoded in DA system in striatum of rodents that appear to adjust their learning rates.

Thank you for pointing to this. We agree that this sentence was hard to parse, and so we now split it in three revised sentences. We also agree with the Reviewer’s interpretation, and would like to thank the Reviewer for their useful literature suggestions which we now added to our discussion.

(7) Figure 7 should use a different color scheme because many of the activations just appear black, and I can't tell whether they are positive or negative. It was also notable in Figure 7A that regions are not visible, including ACC, which is typically thought to encode prediction errors in such paradigms. It would probably be useful for the authors to include a table of all clusters exceeding multiple comparisons correction and to on differences to other work examining absolute prediction errors. ACC does appear on the second trial, which made me wonder whether there were changes in the prediction error coding from first to subsequent trials.

Thank you for pointing this out. We now revised our color scheme which we agree makes it much clearer now. Although the ACC is frequently implicated in prediction error–related signals (e.g., Behrens et al., 2007), models suggest that ACC responses more strongly reflect unsigned prediction errors, surprise, or the need for control and model updating (Alexander & Brown, 2019; Hayden et al., 2011; Silvetti et al., 2018). In our task, ACC activity only emerged on the second trial, when participants had formed an initial estimate and prediction errors could meaningfully signal the need to update internal models or control settings. We now added a to the Discussion highlighting this distinction and relating our findings to this prior work emphasizing prediction errors and control-related signals in ACC.

(8) The authors suggest that fast learning would presumably occur in a neural activation space, whereas slow learning would occur through weight adjustments. This makes sense, but activity-based dynamics have been suggested to do rapid adjustments by encoding a "latent state" though (Razmi 2022 j neurosci) -- and such a latent state has been shown in OFC (Schuck etc)... but here OFC is more implicated in the slow learning. I am curious about whether authors could on this a bit in the discussion.

Thank you for bringing up this interesting question. We can only speculate but a crucial factor is on which level of resolution tasks states operate. On the one hand “detailed” trial-level states are needed that map a specific sensory input onto a specific latent state and its value. Such states would change quickly, possibly through activation dynamics, and are in line with how they have been operationalized in Razmi or Schuck etc. On the other hand, successful task performance also needs “higher level” states that describe entire task phases or full tasks, as in the present experiment. Due to the different speeds of learning, it appears plausible that these would be learned with synaptic changes. We expand on this in the discussion as follows:

“Our finding extends this notion by suggesting a link between OFC and meta learning, wherein meta-learned information becomes encapsulated in task states (Hattori et al., 2023; Moneta et al., 2024). Consistently, OFC has been shown to represent task states (Moneta et al., 2024; Stalnaker et al., 2015; Wilson et al., 2014). While earlier evidence shows that the OFC represents concrete aspects of task states, such as task-relevant stimulus features (Schuck et al., 2016), we hypothesized that the OFC also represents more abstract aspects, such as learned, environment-specific learning rates. Indeed, we showed that the central OFC gradually came to represent these environment-specific learning rates (or the environment-specific statistics that drive them). While previous work speculated that these different levels could have different neural underpinnings (Sharpe et al., 2019), our findings indicate OFC might signal states on multiple levels. This does not imply identical learning dynamics; fast-changing trial-specific states might be learned through activity dynamics, while higher-level contextual states could involve synaptic plasticity.”

(1.9) Also, as a more minor point in the same section, the sentence about blocking synaptic plasticity in OFC sounded interesting, but should have a reference.

Thank you for noticing, we now added the reference (Hattori et al., 2023).

**Reviewer #2 (Recommendations for the authors):**

(1) Additional links to prior literature: In terms of prior work in which there is something akin to more "global" adaptation, some examples of potentially relevant prior work include:

McGuire, Nassar, Gold, & Kable (2014) Neuron

D'Acremont & Bossaerts (2016) Cerebral Cortex

Lee, Gold, & Kable (2020) Decision

Bakst & McGuire (2021) JEP: General

Bakst & McGuire (2023) Cognition

We would like to thank the reviewer for pointing us to these different literature suggestions which we agree help us contextualize and discuss some of our findings better. We now refer to McGuire et al. (2014) when discussing the fMRI results, and d'Acremont & Bossaerts (2016) when discussing potential alternative strategies in the high noise environment (the Reviewer’s last point). Finally, we integrated the clearly relevant works of Bakst & McGuire (2021; 2023) and Lee et al. (2020) in our discussion of meta-learning different adaptive strategies.

(2) Individual differences: Though not always the focus of work on predictive inference, one common finding has been that there are pronounced individual differences in behavior (see, e.g., coefficients in Figure 2 in Nassar et al. 2019 eLife, or Figure 2 McGuire et al. 2014 Neuron, or Bakst & McGuire 2023 Cognition). There appears to be substantial variability between individuals in your data as well (i.e., Figure 2B, 4B, and the modeling figures). It would be interesting to see some direct exploration of this variability: baseline learning rate appears to differ between participants to a large extent, does their rate of adaptation (across trials within a block) also differ? Does their metalearning occur at different rates (in fact, do some participants not show evidence of appropriate meta-learning at all)?

Relatedly, your computational modeling approach fits the six candidate models hierarchically, and therefore the reported results show the overall best fit for the group. It might be worthwhile to determine whether individuals have different best-fitting models. This could be another way to characterize the variability between individuals.

In concert with this, it could be a useful complement to determine whether either the strength of the OFC neural similarity results or their time course reflects aspects of behavior. Put another way, is it the case that not only does OFC activity and behavior both come to reflect task structure, but that these changes happen to a similar extent and over a similar time course across individuals?

We agree it would be highly interesting to investigate meaningful individual differences in both fast and slow adaptations in learning rate. However, our sample was not set up and is underpowered to conduct such analyses. In response to a similar by Reviewer 1, we did run correlational analyses between differences in learning rate, performance accuracy, and the responsiveness of the OFC. However, none of these analyses yielded a significant effect. We decided to not include these results in the paper, for reasons of statistical power, but we report them in Author response image 1.

(3) fMRI:

(3a) The primary finding in OFC is restricted to the central OFC. The manuscript would benefit from additional explanation regarding this specific subregion.

Thank you for bringing up this important distinction. In the discussion we now clarify as follows:

“This difference reflects our hypothesis that the OFC represents the latent states in a cognitive map of the task (Wilson et al., 2014; Schuck et al. 2018; Knudsen & Wallis, 2022; Moneta et al, 2023), which are expected to activate as soon as the agents can infer which task state it is in. Several studies have identified such “partially observable” task states in the medial OFC (Schuck et al., 2016; Bradfield et al., 2015; Wimmer et al., 2019; Tan et al., 2025), in line with the region identified here (but see e.g., Öngur & Price, 2000, for important anatomical distinctions between medial and lateral OFC and Tan et al., 2025, for an example of related functions in lateral OFC).”

(3b) Though the main clusters visible in Figure 6 are the occipital and OFC clusters, there appear to be others. Did other clusters indeed rise to statistical significance in the whole-brain analysis? If so, is there a reason they aren't included or discussed?

All clusters visible in Figure 6C survived FDR correction. However, we refrained from interpreting these other clusters, because we had no prior hypotheses about them like we did for the OFC.

(3c) Why do you posit that the ventral striatum becomes less sensitive to RPE on the second trial over time? And why is the ventral striatum only sensitive to RPE in the low noise environment generally?

We reasoned the ventral striatum should be more responsive to more positive reward prediction errors. While we further assumed this response could be modulated by both time and environment, we would like to emphasize that we had no specific hypotheses about the direction of this modulation. We now also make this clearer in the manuscript. This being said, we believe both the pattern that its responsiveness to the second trial decreases over time, and the pattern that it was most sensitive to the low noise environment, can be considered fitting with its broader involvement in coding behaviorally relevant reward prediction errors. Namely:

First, we believe that as the participants learn more about the global reward structure of the task, they should obtain a better understanding of the fact that, per round, all crabs always center around a fixed mean. Therefore, the first RPE is most behaviorally relevant, and every later RPE has an exponentially decreasing relevance. As participants obtain more experience with this aspect of the task over time, the VS should show a lower responsiveness to the second RPE over time.

Second, as participants learn more about the local differences between the three different environments, they should learn that especially in the low noise environment, RPEs are most behaviorally informative. That is, in this environment it makes most sense to have a high learning rate and thus let the RPEs substantially inform the placement of the cage on the next trial. Accordingly, participants showed that the ventral striatum was most responsive to RPEs in these environments.

(4) Methods

(4a) This section could generally benefit from some proofreading.

We now proofread the method section.

(4b) The main results text states that 49 participants performed Experiment 1, while the methods section reports 50 participants. Which is correct?

(4c) Following this, on page 8, statistical results are reported with a df = 49 (which would be appropriate only if n=50).

The correct sample size was actually 50, we adjusted the text and degrees of freedom where incorrect accordingly (note: only text is in track changes, but degrees of freedom were also changed accordingly).

(4d) Additionally, I am a bit surprised by the Experiment 1 findings that learning rates on the second trial were significantly different between low and high noise conditions, in that the effect size found using all trials was stronger than both the first half of trials (no significant effect) and the second half (significant but weaker than all trials). Are these all the same type of statistical test? Double-checking the statistics might be worthwhile.

It is not the effect size that is larger across the full experiment, but the t-statistic. This is possible because a t-statistic depends on both effect size and noise estimate, and the latter is smaller with more data.

(4e) The methods and results both state that the five crabs always emerged from one position in the sand. How were the locations of the crabs selected relative to this position? Looking at Figure 1C, it looks like the crabs spread out unevenly, and that the single position they emerge from is not necessarily at the center of the crab locations.

The crabs did indeed spread out evenly. However, we can see how the graphic in Figure 1C can be confusing, as two crabs are shown to be caught, which breaks the symmetry of the dispersion (because some crabs can run away after the even spreading phase, see Methods). We emphasized the even spreading more clearly in the new version of the paper. We think the flow of events will be much clearer with our newly added animation (Video 1).

(4f) The methods section states that the crabs "spread out to cover the same proportion of the screen width as the cage (18.75%)" (page 23). The corresponding visual in Figure 1C appears to show something different.

This looks different because the graphic illustrates the last 500 msec, where crabs can run away (see also response to 4e, and the novel animation that was added).

(4g) Information on the timing of the trials would be useful to include in Figure 1C or similar.

The reader can find this information in the Methods section. We chose not to include it in the caption to avoid information overload.

(4h) The methods section specifies that there was a 3-7s ITI after the first and second trials of each block. How was the ITI selected for each trial? Were there ITIs between the other trials? If so, what were they?

The ITIs were selected from a truncated exponential distribution. This selection was not random, but rather a distribution was carefully constructed for each environment (and event of interest: boat presentation, first trial of each block, second trial of each block) separately to ensure that enough longer ITIs were selected for each environment (and event of interest). Of course, the order in which the ITIs were used across blocks, was random. The same approach was used to determine the duration of the presentation of the boat at the start of each block. There were no ITIs after later trials.

(4i) Please provide a link to the data and analysis materials on OSF in the text.

We now provide a link to the data and analysis materials in our methods section.

(4j) In the methods section, there are some references to information provided "below" (page 26: "The two approaches resulted in different posterior densities (see below) for estimate uncertainties, but in similar posterior densities (see below) for learning rates..."). Where in the paper is this referencing?

We indeed did not detail this further as we considered it not further relevant to our main study, and now removed the references to “below”.

(4k) The methods section specifies using uniform priors between the lower and upper bounds of the relevant parameters. This seems likely to be 0 and 1, but should be listed explicitly.

Thank you for noticing. We now added this to our manuscript.

(4l) For parameter recovery, correlations are provided to indicate effective recovery. These correlations are indeed high and suggest excellent recovery, but correlations wouldn't reveal if there was systematic over- or underestimation occurring. It might be useful to provide some visualizations of the parameters and their estimates to speak to this potential issue.

We now visualize the parameter recovery results in Author response image 2, which show that, indeed, there was a slight underestimation of the decay rates, but not the learning rates. Importantly, our main analyses and results all pertain to the learning rates, and we never made hypotheses or conclusions about the decay rates.

**Author response image 2.**

(4m) The methods section ends with a reference to a reward localizer (page 32). This localizer doesn't appear to be mentioned/used elsewhere.

Indeed. We implemented the localizer because we wanted to independently identify reward processing areas. However, this localizer did not succeed in localizing a reward area (no significant results), possibly due to the fact that (1) it was performed by the end of the experiment when participants may have been fatigued, and (2) there was no learning component in this localizer task. For these reasons, we did not use it after all.

(5) Analysis:

(5a) Did you consider fitting a Bai model that only allowed for environment-specific initial learning rates (with a non-environment-specific decay rate)? Given that the data (e.g., Figure 2, Figure 4) seems to support differences in initial learning rate but not necessarily a difference in the rate of change, it might be worthwhile to see whether a model like that fits best.

We now fitted this extra model, which we called the semi-environment-specific Bai model. See Author response tables 1 and 2 for result in experiments 1 and 2, respectively for the results. This new model has the best (in Experiment 2) and second-to-best (in Experiment 1) LOOIC. In a way, this is not surprising, because the model formulation is entirely based on the data. We think that we can draw the same substantive conclusions with or without this extra model, so for simplicity we did not include this new model in the paper itself.

**Author response table 1.** 

Model	LOOIC	SE	DLOOIC	△SE
Environment-specific Bai model	28735	404	0	0
Non-environment-specific Bai model	28582	441	153	65
Environment-specific Rescorla–Wagner model	28436	398	299	67
Non-environment-specific Rescorla–Wagner model	28172	440	563	112
Environment-specific Kalman filter	27853	493	882	211
Non-environment-specific Kalman filter	27698	521	1037	211

Note. Models are ranked in descending order according to how well they fit the data. LOOIC refers to a model’s approximated expected log pointwise predictive density. Higher values indicate higher out-of-sample predictive fit. SE refers to the standard error of a model’s LOOIC. ∆LOOIC refers to the difference between a model’s LOOIC and the top ranked model’s LOOIC. ∆SE refers to the standard error of the difference between a model’s LOOIC and the top ranked model’s LOOIC.

**Author response table 2.** 

Model	LOOIC	SE	/_\LOOIC	DeltaSE
Environment-specific Bai model	34725	142	0	0
Non-environment-specific Bai model	34682	158	43	34
Environment-specific Kalman filter	34592	160	133	42
Environment-specific Rescorla–Wagner model	34567	136	158	32
Non-environment-specific Kalman filter	34523	174	202	49
Non-environment-specific Rescorla–Wagner model	34434	154	291	51

Note. Models are ranked in descending order according to how well they fit the data. LOOIC refers to a model’s approximated expected log pointwise predictive density. Higher values indicate higher out-of-sample predictive fit. SE refers to the standard error of a model’s LOOIC. ∆LOOIC refers to the difference between a model’s LOOIC and the top ranked model’s LOOIC. ∆SE refers to the standard error of the difference between a model’s LOOIC and the top ranked model’s LOOIC.

(5b) If part of the goal is to investigate whether there is a distinct local change in LR between conditions (dependent on prediction errors), then there might be more direct ways of doing so as a complement to the modeling approach. One potential way could be to visualize the LR or change in LR as a function of PE.

We agree that it’s beneficial to use a direct (model-free) approach to represent learning rate as a function of condition; that is also part of our approach. For example, see Figures 2, 4, which shows learning rate as a function of condition, but in a model-free manner. We think learning rate as a function of prediction error is less informative, because the idea is that prediction error can (in Kalman-filter terminology) be indicative of either noise variance or process variance, and participants are able to distinguish between them. This is also why we constructed the conditions in such a way that on the very first trial, prediction errors were on average the same across conditions. The fact that participants did respond appropriately to prediction errors on the very first trial (i.e., larger updates or learning rates in the low noise condition), suggested they are able to assign the prediction error to process variance (in the low noise condition) versus noise variance (in the high noise condition).

(5c) In addition to looking at the evolution of LR across trials within a block separated by task epoch (i.e., Figure 2C-D & Figure 4C-F), the structure of the task would lend itself very nicely to visualizing the evolution of the second trial LR on its own across instances. This could provide additional insight into the meta-learning process.

We thank the reviewer for this interesting suggestion, which was also raised by Reviewer 1. We now calculated the learning rate in a sliding window of 20 trials (i.e., trial x to x + 19), and provide revised figures for each experiment separately (Fig. 2 and 4, respectively).

(6) The environment-specific Bai model appeared to become less good at capturing participant behavior with increased environmental noise. Why do you think this is?

We thank the reviewer for raising this point. In this environment, individual outcomes are considerably less indicative of the latent mean, which may reduce the usefulness of the trial-by-trial, prediction-error–driven learning-rate adjustments that we see in the other environments. Under such extreme conditions of variability, people may rely less on delta-rule updating and more on alternative strategies (D'Acremont & Bossaerts, 2016; Reynders et al., 2026), such as exploratory adjustments or heuristics that are not explicitly captured by the Bai model but also outside the scope of the present paper.
